# Plant breeding and diversity: A troubled relationship?

**DOI:** 10.1007/s10681-018-2192-5

**Published:** 2018-06-19

**Authors:** Niels P. Louwaars

**Affiliations:** 1 Plantum, Netherlands Association for Plant Reproductive Materials, Gouda, The Netherlands; 2 Department of Law and Governance, Wageningen University, Wageningen, The Netherlands

**Keywords:** Genetic resources, Diversity bottleneck, Plant breeding, Genetic diversity, Biodiversity policy, Seed regulations, Trait breeding, Participatory plant breeding, Multiline, Nagoya Protocol

## Abstract

Plant breeding collects, induces and rearranges genetic diversity followed by selection. Breeding may contribute to diversity in farmers’ fields or significantly reduce it. History has numerous examples of both. The diversity of many crops have gone through domestication, dispersal and modernization bottlenecks. Between these major decreasing processes, diversity has picked up through different evolutionary processes, and plant breeding affected by policies. Major negative effects of plant breeding on diversity have been recorded following the modernization bottleneck, but alternative breeding strategies have come up as well, both in the formal system and in the interphase between formal and farmers’ seed systems. Multiline breeding and participatory plant breeding are introduced as examples to also analyse effects of current developments in technology and policy. This paper intends to shed some light on the questions: how will current developments in technology and policy affect crop genetic diversity? Are we heading for a new bottleneck—either a molecular or a policy bottleneck, or a combination of both? Or could the future become more diverse? We look at the relationship between breeding, policies, and crop genetic diversity in farming systems with a birds-eye view. Notably because of current policy trends we warn for a new diversity bottleneck.

## Introduction

Plant breeding collects, induces and rearranges genetic diversity followed *by selection.* The balance between these diversity enhancing and reducing forces determines the outcome in terms of gain or loss of diversity at the end of the breeding process. However, the effect of breeding on diversity in farmers’ fields is more complex. The seed systems that the breeding operation feeds into and various policies greatly affect the outcome in terms of diversity. Can an analysis of past processes that shaped current diversity levels inform us about the future? By reviewing the grand historical developments we analyse the trend leading to the modernization bottleneck, including the roles of plant breeders and policy makers. We also introduce two initiatives by breeders to illustrate processes that go against the trend of reducing diversity.

Technological developments in plant breeding are ongoing, and also at the level of policies significant developments occur at the moment. Can trends since the modernization bottleneck be extrapolated, will these current developments give rise to another bottleneck, or will they affect crop genetic diversity in different ways in the coming decades?

### Domestication and dispersal bottlenecks

On a historic scale many crops have gone through a defined process that shaped the genetics and diversity of our current crops. The ‘big picture’ is that during domestication only a limited part of the species’ diversity was selected to serve the needs of early farmers. The cultivated crop thus has a limited diversity compared with the wild species that it originates from Tanksley and McCouch (1997). This has been framed as the domestication bottleneck in genetic diversity. Such bottlenecks have been particularly severe where particular mutations or polyploidization created essential characteristics that made the plant a useful crop for mankind (Lelley et al. 2000). After such initial bottleneck, diversity levels of crops may have increased through a combination of natural and intentional selection in the various farming systems and microclimates where they were grown and through introgression of genes from the wild population.

Many crops were introduced in areas outside their centre of domestication. During this process a larger or smaller part of the diversity of the cultivated crop was used as the genetic base in the ‘new’ continent. This created a ‘dispersal bottleneck’ (Zeder et al. 2006). Recovery of diversity following such dispersal over large distances has likely been more limited as introgression with wild populations was not possible—assuming that the species itself or a related wild species does not occur in that new environment. Evolution of crops in isolation from their ancestors would depend only on mutation and selection in the new farming systems, and possibly through occasional or intentional new introductions from the region where the crop was initially domesticated. With time, secondary centres of diversity could develop where specific ‘new’ diversity arose (Pickersgill 1998).

The above processes occurred in the farmers’ seed systems creating the landraces that formed the basic diversity that scientific breeders used as their source materials. It must be assumed that early plant breeding in field crops concentrated on selection among and within such landraces. Even though sexuality of plants was already published by Camerarius in 1694, it was hotly debated well into the nineteenth century (Žárský and Tupý 1995), thus delaying intentional cross-breeding in field crops.

### Modernization bottleneck

#### Breeding and seed production

Even though early examples show that crosses with materials from other regions created useful advances in wheat as early as the early nineteenth century (Louwaars and Burgaud 2016), systematic cross breeding in field crops started only in the 1880s together with the ‘invention’ of line selection and pedigree selection in Svalöf, Sweden (Nilsson 1898).

Cross breeding greatly intensified after Mendels laws of heredity were rediscovered in 1900 (De Vries 1900a, b). Crossing different plants produces genetic diversity that can be selected in. However, the more advanced selection methods that were developed at that time as well, made it possible to develop more uniform new varieties, which replaced diversity embedded in the landraces that farmers had used to grow.

Plant breeding intends to combine as many ‘favourable traits’ as possible in one genotype or maximise the presence of such traits in one population. Diversity within the variety is thus reduced. Plant breeding itself is a major driver of uniformity in a farmers’ field. Also other pressures played a role towards more uniformity: early mechanisation, requiring more uniform crops and large scale processing of agricultural produce requiring standardised product qualities.

Even though selection leads to reduced diversity within a variety, diversity among varieties can still be significant. However, efficiency in breeding is an important driver of reducing such diversity: both for public and private breeders it is important to have a sufficiently large ‘recommendation domain’ for their new varieties (Chambers 1985; Evenson et al. 1979). For private breeders it is important because their business model is based on the quantity of seed of their new variety that can be sold; public breeders have to make sure that public funds benefit as many farmers as possible. In addition, the seed production organisation determines the number of varieties that can effectively be multiplied. A seed producer managing too many varieties at the same time is bound to run into problems with his production planning and seed marketing operations. Breeders in a competitive market not only have to breed the right varieties, they also have to get them to the market before their competitors have reached similar yield levels or other important improvements. This led in the 1960s in The Netherlands to a situation where all wheat breeding companies made crossed between the two best performing varieties at any particular year. Speed of selection and maintaining an appropriate mix of other breeding goals determined the success of the company. Breeders could not easily afford to make crosses with less related materials to enhance diversity in their breeding programme. That task was commonly left to the public sector as this was considered pre-competitive research.

The modernization bottleneck occurs in different crops and geographies at different moments in time. It is best illustrated by the Green Revolution where the famous shortstraw rice variety “IR 8” became the dominant variety in large parts of south and southeast Asia, and in Latin America following its release in 1966 (Lipton and Longhurst 1989; Evenson and Gollin 2003). Such semi-dwarf rice and wheat varieties replaced a wide variety of different landraces in many countries. The success of the early Green Revolution varieties contributed significantly to the recognition that plant genetic resources need to be preserved (Harlan 1992). This example also shows a major change in public plant breeding: globalisation. Whereas breeders had used to operate at the local or national level, international cooperation in breeding during the Green Revolution not only made genetic resources from a wider geographical area available to breeders (increasing diversity available to individual breeders), it also led to breeders in different parts of the world using the same materials as parents for their local breeding, thus—initially at least—reducing diversity among breeding programmes worldwide.

#### Policies

Public interest in seeds led from the 1860s onwards to the establishment of seed laboratories to independently assess the quality of seed following calls from farmers. The further modernization of agriculture and the use of modern uniform varieties furthermore led in several countries in the early twentieth century to certification of varietal identity and purity, and the establishment of lists of recommended varieties. Registration of variety names and seed certification were subsequently codified in many countries, which established international standards for seed testing methods (ISTA 1924) and seed certification (AOSCA in 1919 and OECD Seed Schemes in 1958). Since seed qualities can often not be observed from looking at the seed, farmers require a reliable label with variety name and quality guarantees. Trueness to labelling became the basis of seed regulations in the USA. The basis of such variety and seed regulations is farmer protection. This increasingly strict regulation of varietal purity furthermore led to the reduction of within-variety diversity. The importance of within-variety uniformity for breeders increased further with the introduction of Plant Breeder’s Rights in various countries from the 1940s onwards, and their harmonisation through the Convention on the Protection of New Varieties of Plants in 1961 (www.UPOV.int). However, Van der Wouw et al. (2013) attribute an observed increase in diversity among varieties in the 1970s to the stimulating effect of the protection of breeder’s rights towards the over-all breeding effort in Europe. The rights provide breeders with a chance to recoup investments in plant breeding. They consider the markedly increased investment in breeding of lettuce an important factor in the increased number of varieties and diversity that they observed. This appears to outweigh possible effects of the trend that the number of breeding companies declined from 1970 onwards. The larger breeding programmes could invest in using a wider array of parent materials in their breeding programmes (Van der Wouw et al. 2013).

The variety registration and release systems, notably the compulsory ones in many countries, limit the number of varieties available to farmers and impact breeding. Release systems that are based on formal multi-location trials that are analysed by standard overyears/location ANOVA calculations, tend to select few varieties with broad adaptation that do well ‘on average’ rather than several with value for more specific uses (Tripp and Louwaars 1998). Various policies thus directly or indirectly affect the diversity within and among varieties in farmers’ fields.

## Diversity after the modernisation bottleneck

Breeders’ responses to the modernization trends and technological developments on the one hand, and existing and evolving policies on the other affect the diversity in the field following the modernisation bottleneck. Various mechanisms evolved to increase diversity, both in breeding programmes and in products of breeding.

### Examples of breeding for diversity

With two examples we illustrate that the limitations of the above mentioned trends have received attention from breeders: the wheat breeding at the Zelder cooperative in The Netherlands, and participatory approaches as developed in the global South.

#### Diversity in commercial breeding

The senior wheat breeder of small breeding company Zelder in the Netherlands in the 1970s pioneered in new ways to combat yellow rust. He saw that the treadmill that the breeders were caught in the gene-for-gene resistance management was about to collapse. He developed two strategies in the late 1970s and 80s in order to curb rust epidemics. First, he developed the first commercial multiline; a variety consisting of 5 lines that differed in their resistance patters but that were otherwise isogenic (Groenewegen 1977). The diversity in the field would significantly slow down the build-up of the fungal populations and the disease would thus remain below damage levels. The concept worked, but it had no impact in farmers’ fields. The variety “Tumult” reached the market in 1980 following some additional years of selection compared to conventional line selection to develop the near isogenic lines. The result was that the yield levels of the competitors’ varieties were already a few percent higher, which caused farmers to rejected “Tumult”. The name was however well chosen, because this new approach created some havoc with the variety registration and seed certification officials because they had to put an effort in fitting this genetically diverse variety in their regulatory systems.

The second approach was to collaborate with the public institute SVP in a programme to introgress resistance genes from related species into elite material and to work on horizontal resistances that should create a lower selection pressure on the pathogen and thus would be effective for a longer time (Parlevliet 1977). The results were positive, but by the time the strategies yielded results the financial position of the company had deteriorated and it was sold. These examples show that breeding for diversity is difficult in crops with low profit margins such as wheat.

#### Diversity in less formal settings

A very different deviation of the standard organisation of breeding is the development of various forms of participatory plant breeding. These were initiated with varying objectives and methods. Witcombe, working mainly in India (Witcombe et al. 1996; Witcombe and Jayavendra 2014) and Ceccarelli working from ICARDA (Ceccarelli and Grand 1991; Ceccarelli et al. 2009) are breeders who realised that centralised breeding programmes cannot sufficiently cater for diverse local needs, especially at the level of smallholder farmers. Such farmers may value yield stability over potential yield; and may have different breeding goals such as straw yield and culinary properties. Centralised breeding may moreover not be able to cater for the diversity of agro-ecosystems in the region that they breed for and the resulting G × E interaction. Participation with farmers should resolve many of these shortcomings of the formal system with a result that many different varieties are selected. On the other extreme of the participatory breeding spectrum, scientist such as Sperling in Rwanda (Sperling et al. 1993) put trained breeders in a supporting role towards farmer-seed specialists who effectively manage plant materials in their local seed systems, but who benefit from support from trained breeders (Sperling et al. 2001). Next to breeding better varieties and maintaining diversity, farmers’ empowerment is an import goal. These participatory forms are built on farmer-field schools and other methods developed in the social sciences. Next to developing better varieties farmers’ empowerment is an important goal.

Both strategies can lead to increasing diversity, both within or among varieties since each participating farmer will select materials that do well in their valley, in their farming system. It is then expected that seed of locally well performing selections move among farmers in the farmers’ seed systems.

### Technological developments

Various technological developments opened up possibilities for increasing diversity in plant breeding. Cytological studies prepared the way for various interspecific crosses, widening the genetic base of plant breeding for several crops. In addition, developments in molecular technologies opened up quite different opportunities. Genetic modification makes the introduction of functional genes from unrelated species possible, but this technology remained limited to very few events that actually reached farmers’ fields.

Marker assisted selection appears much more important as it greatly facilitates the practical use of more genebank materials in breeding. Introgression of traits from distant materials or even wild relatives became much cheaper and—commercially more important—much quicker. The use of such materials thus potentially widens the effective genetic base of crops in practical breeding. More recent developments such as cis-genesis, gene editing and the use of full genome sequences—including in genebank management—open up additional opportunities.

### Impact of policies on breeding for diversity

Policies have a marked impact on the different examples of breeding for diversity and the technological developments introduced above.

Multiline breeding is interesting from an agro-ecological point of view but proved commercially difficult to implement. An important set-back was that in the European setting of compulsory variety registration and seed certification, multilines do not fit in the variety concept on which the regulations are based (Zeven and Waninge 1985). Also the uniformity requirement of plant breeder’s rights creates problems. This contributed to the time lost and subsequent commercial fiasco of ‘Tumult’.

Also participatory plant breeding creates challenges for the regulators. Varieties selected in such settings are rarely uniform and do likely not pass national multi-location trials for varietal value for cultivation and use. The farmers on the other hand, may not even want the varieties to be registered since they will share it with their neighbours and will allow the selected varieties (populations often) to evolve in their specific environment. They also unlikely are able to pay for the registration and release trials. Also obtaining protection on such varieties is often not a farmers’ goal (Salazar et al. 2006).

#### Seed laws

So, seed laws can run counter to such initiatives to increase diversity through breeding. The European Union has introduced the concept of ‘conservation variety’ to create policy space for the marketing of seed of old, heterogeneous, varieties with specific adaptation and qualities (Bocci 2011). This opening cannot be used for new products of participatory breeding. Recent discussions in Europe on organic regulations may create options to allow heterogeneous varieties for certain crops and uses. Dropping all limitations on varietal uniformity and identity could, however, create opportunities for fake seed in the conventional seed markets, which would damage farmers’ interests. Better analysis of the diverse needs could lead to a framework for both seed laws and breeder’s rights laws with enough policy space for a diverse seed system landscape (Louwaars et al. 2013).

#### Regulating technology

Policies also impact the use of technological opportunities. Genetic Modification has been regulated in almost all jurisdictions, creating great financial and legal complexities for parties that want to use the technologies, and especially those that want to put their products in farmers’ fields and in food or feed value chains. The result is that the number of GM-events that are used in agriculture and the number of companies involved are very limited. The fact that regulations greatly differ in different countries adds to the complexity. Even though technically, modification can add diversity, the effect has been limited in practice as the complexities of deregulating GM-traits resulted in very few traits that add to diversity. It is also likely that even though these traits have been bred into a locally adapted varieties and hybrids, the quick uptake of the new technology by farmers has led to reduction of diversity in the field. Such regulatory involvement is not encountered in use of marker assisted breeding technologies described in the previous section. These are widely used by both public and private breeders and researchers of many crops, including with the aim of widen the genetic base of breeding.

The effective genetic base of breeding is also affected by the actual access to genetic resources, which was supported initially by the breeder’s exemption in Plant Breeder’s Rights, and the formalisation of the concept that genetic resources are a “heritage of mankind” in the “International Undertaking for Plant Genetic Resources for Food and Agriculture” (FAO 1983). The situation changed when the UNCED Conference in Rio de Janeiro assigned national sovereign rights over its genetic resources. According to the Convention on Biological Diversity (CBD 1993), countries have the obligation to conserve and promote sustainable use, and can make access to such resources subject to mutually agreed terms and prior informed consent in order to secure equitable sharing of benefits. Most ‘provider’ countries have not been able to put in place effective and efficient mechanisms to implement these rights and obligations. In order to reduce risks that reduced availability of genetic resources would impair global food security, the countries, within the scope of the CBD agreement formulated the International Treaty on Plant Genetic Resources for Food and Agriculture (FAO 2001—in force 2004) with the aim to facilitate exchange under multilateral systems for access and benefit sharing.

Even though collection of agricultural genetic resources appears to have been reduced during the first 2 decades of the CBD, exchange of materials of the main food crops has generally been ongoing.

## Are we heading towards a new bottleneck?

Since the start of the twenty-first century, both technology and policy keep advancing at a rapid pace creating various dilemmas in relation to diversity in crops. How technology will change the face of plant breeding and its effect on the diversity in farmers’ fields is yet unknown; some trends in policy are clearer with regard to their effect on breeding—and especially those programmes that intend to broaden the genetic base of breeding and to putting diversity in the field. Looking ahead in time means that what follows is speculative by definition. The intention of the analysis is, however, that it may influence the same policy trends that we are describing.

### From plant breeding to trait breeding: molecular technology bottleneck or opportunities?

Plant breeding has always been focused on a number of clear objectives that breeders were selecting for, but for less important traits and in non-coding DNA diversity within and especially among varieties can be significant. Such ‘chance combinations’ add to diversity. Where molecular techniques that support selection seem to have increased diversity through the wider use of wild relatives, they also made it possible to increasingly precise removal of unwanted traits in crossing populations. Technological developments have moved on so the question is what effect the latest breeding methods may have on diversity?

Where markers have enhanced opportunities to use wild relatives, gene editing may significantly reduce the need to do that since it provides efficient ways to create and investigate minor changes in any gene. For example, the increasingly systematic creation of point mutations in a resistance gene will yield ‘new’ alleles or may allow the use of alleles present in the known diversity without having to cross-breed dragging along all kinds of unwanted traits. This may mean that a flaw in a good variety can potentially be corrected in the near future without the need to create a whole new variety.

In some crops, such as maize, we can already speak of trait breeding. Introducing ‘new’ diversity in the populations from which the inbreds are created is already increasingly complex, but current breeding based on the detailed knowledge of the genome allows such levels of precision in the relation between genotype and phenotype that linkage drag is minimised (Peng et al. 2014) so that there is little room for ‘chance combinations’ that were always introduced with crossing different parents. Gene editing and the use of sequence information in genome-wide selection will further add to the precision.

The developments in gene editing may also create new opportunities for supporting diversity! If indeed, different alleles can be created or copied from existing diversity by changing some base-pairs, gene editing may create new opportunities for the development of multilines. If a variety of alleles of a resistance gene could be created in a single variety, it would be much quicker to create near-isogenic lines than in conventional cross breeding at the time “Tumult” was developed. It would be interesting to create a proof of concept of this approach.

Technological developments may thus create ‘new’ diversity in the target traits, but what the total outcome will be in terms of diversity is not clear. This outcome will also depend on the policies associated with these breeding methods. The importance of gene editing methods in plant breeding in practice depends highly on policy decisions with regard to the regulatory frameworks that may apply to the products of such methods. Several countries are currently debating whether such methods fall under their current definition of genetic modification, and whether resulting plants may have to be regulated under GM or other rules.

### From promotion to avoidance of use of genetic resources: Towards a policy bottleneck?

At the level of rights on plants, major impacts of current developments on diversity may be expected. Plant breeder’s rights that allow anybody to use of protected varieties for further breeding, has been identified as having a positive impact on breeding and thus diversity among varieties. Two developments may, however, obstruct that freedom: patent and biodiversity laws.

The patent system did not significantly influence plant breeding until the latter part of the twentieth century. Court decisions (in the USA from 1985 onwards), and policy changes (in Europe in 1998) made it possible in different jurisdictions to obtain patent protection for a plant variety (predominantly in the USA) and on plants expressing particular traits (in a larger number of countries). Plants that fall under the scope of such (utility) patents are not automatically free for further breeding. Breeders have opposed to this trend in several countries. This culminated in Europe to the introduction of a ‘limited breeders exemption’ in national and EU patent laws and in June 2017 the European Patent Office decided not to provide patents anymore on naturally existing traits, formulated as ‘products of essentially biological methods’ (European Patent Office 2017a, b). This followed the views of the European Commission that it had never had the intent at the time of formulating the 1998 law to provide patents on such products (European Commission 2016). It appears though that products of technical processes will continue to be patentable when they are sufficiently inventive and new which indicates that following the development of the latest breeding methods, access to genetic materials may still be limited by patents. The wider the range of materials that breeders want to use in breeding for diversity, the more effort they will have to put in investigating the status of that material. This is a challenge for formal breeders, but even more so in participatory settings, where the required legal counsel may not be readily available. In this view, attempts by breeders to facilitate the granting of licenses, such as the International Licensing Platform created by vegetable breeders (www.ilp-vegetable.org) are commendable. Unlike the sovereign rights on genetic resources, discussed hereunder, patents expire (after 20 years in most jurisdictions), which means that the technologies and materials become part of the public domain. Biodiversity rights are in principle eternal which could potentially impact breeding for diversity much more.

The second trend is the implementation of the rules of the Convention on Biological Diversity (CBD). The impact on practical breeding has not been devastating during the first 2 decades. A more recent development, putting responsibilities to the user rather than the provider under the “Nagoya Protocol*”* (CBD 2010—entering into force 2014), could greatly complicate both breeding research and practical breeding. The implementation of this Protocol is currently under debate in many countries. (Draft) laws and implementation guidelines appear to create situations that resemble eternal patent rights as users of the resources must at all times have a license from the authorities of providing countries and be able to prove that the parents that have been used in breeding have been obtained in accordance with such license. This could—similar to patents on different traits in one plant—apply to authorities from different countries from where plant materials have been obtained. This puts a vast administrative burden on breeders and creates uncertainty especially since some countries prepare to include retroactivity of claims, or may put obligations on due diligence also the use of released varieties, thus undermining the UPOV provisions on the breeders exemption. Debates at the international level also extends to whether benefit sharing is also required on the use of digital sequence information in breeding, which will create additional complexities and uncertainties.

These developments create some important dilemmas: complex benefit sharing arrangements will reduce the sustainable use of genetic resources in breeding, which is also an objective of the CBD. Will the change in the burden of proof on benefit sharing towards the user also introduce a burden of proof on countries with respect to their obligations to promote the sustainable use of their genetic resources and their conservation efforts? Do negotiators realise that complex administrative measures in relation the benefit sharing affect smaller breeders more than larger breeding companies that have more administrative and legal capacities, thus reducing the number of breeding programmes further? How do benefit sharing arrangements on digital genomic information affect the current trends towards open data?

More important for this paper is the question what such restrictions will mean for breeding in formal and alternative settings aiming at increasing diversity? It is likely that overly complex and expensive arrangements for benefit sharing may stimulate breeders to limit their work within the gene pool that is unrestricted (notably the materials that they had inhouse before the coming into force of regulations). This will not support breeding for diversity. Higher diversity in output of breeding requires more diversity at the input level. When rights—either private (patents) or national sovereign (biodiversity)—rest on more sources of plant breeding, then using a wide array of genetic resources becomes increasingly difficult. These rules will also make alternative breeding strategies and alternative institutional arrangements such as participatory breeding in informal seed system settings completely impossible. Tracking and tracing genetic resources to operationalise benefit sharing is impossible in a participatory settings aiming at creating populations that are allowed to further evolve under different farming conditions. Such breeding strategies cannot go hand in hand with administrative arrangements required by the new policies. It may be counter intuitive that biodiversity laws will block breeding for diversity, but current regulations seem to do exactly that.

## Conclusion

Plant breeding and diversity have a complex relationship. The role of breeding to combine as many ‘positive’ traits in one plant or one population by definition reduces diversity within a variety. At the same time, breeders can introduce important levels of diversity in farming systems. Plant breeders and seed producers operate in a complex policy environment and this paper shows that regulations can have a significant impact on diversity produced and used.

Crops have gone through different bottlenecks with regard to diversity and breeders have had a significant role, especially in contributing to the modernization bottleneck. At the same time, breeders have ventured into alternative breeding methods and arrangements with the effect that diversity could increase. Breeders also respond to policies which may affect the diversity that they produce. When we look ahead beyond the modernization bottleneck and the trends that increase diversity to some extent, we see some risks and opportunities.

A molecular technology bottleneck may appear in plant breeding when gene editing methods support the trend from plant breeding to trait breeding. This trend does however not necessarily affect alternative breeding strategies to be applied or developed in parallel and gene editing may even be used to increase genetic diversity among varieties and within, such as multiline-breeding. Such strategies will require different incentive structures and may require targeted openings or—better—interpretations of current seed laws.

However, a ‘policy bottleneck’ is more likely to develop, when (time limited) patents and especially (eternal) rights on genetic resources will require complex administrative and legal procedures to negotiate access, arrange for benefit sharing, and resolve conflicts. This will reduce the practical availability of diversity in breeding and will reduce possibilities for smaller operators in formal breeding. It is likely to affect explicit arrangements for breeding for diversity even more, and potentially outlaw breeding methods where tracking and tracing is impossible, such as in most participatory breeding strategies.

This paper raises concerns and calls for policy makers to take these into account and find solutions to the challenges that a new bottleneck will create for the sustainability of agriculture.
